# From Dugout to the Mound: A Tale of Platelet-Rich Performance

**DOI:** 10.7759/cureus.50600

**Published:** 2023-12-15

**Authors:** Rock P Vomer, Daniel P Montero, Shane Shapiro, Emma York, Sara Memon, Chris Fungwe, George G. A Pujalte

**Affiliations:** 1 Department of Family Medicine, Avance Care, Raleigh, USA; 2 Department of Family Medicine, Mayo Clinic, Jacksonville, USA; 3 Department of Orthopedic Surgery, Mayo Clinic, Jacksonville, USA; 4 Department of Family Medicine, Prisma Health University of South Carolina, Columbia, USA; 5 Departments of Family Medicine and Orthopedics and Sports Medicine, Mayo Clinic, Jacksonville, USA

**Keywords:** ulnar collateral ligament, rehabilitation, physical therapy, platelet-rich plasma, conservative management, baseball pitcher, ucl tears

## Abstract

Ulnar collateral ligament (UCL) tears of the elbow are prevalent injuries among throwing athletes and are associated with excessive or repeated valgus forces at the elbow. We present the case of an 18-year-old male baseball pitcher with an 18-month history of progressive right elbow pain, notably worsened during his fastball pitching. Clinical assessment revealed tenderness with dynamic stressing of the right UCL. Imaging analyses, including magnetic resonance imaging (MRI) and dynamic ultrasound, confirmed a high-grade partial tear of the UCL at its origin. Non-operative management was pursued, which included an ultrasound-guided platelet-rich plasma (PRP) injection and intensive physical therapy. Follow-up evaluations at six and 12 weeks demonstrated a noteworthy improvement in subjective pain descriptions and structural healing of the UCL. After the patient completed a therapy and rehabilitation program, throwing activities at full strength were able to be resumed. This case underscores the potential efficacy of conservative approaches in handling UCL tears with the inclusion of PRP as a viable treatment option.

## Introduction

Ulnar collateral ligament (UCL) tear is a common injury sustained by overhead athletes that results from repetitive valgus stresses on the elbow joint [1]. It’s becoming a more prevalent injury as baseball pitching velocity has increased, especially among youth baseball players [1]. Recent statistics indicate an annual incidence of elbow injury ranging from 2.3% among adolescent pitchers to as high as 40.6% in youth pitchers, with a peak age range of 15 to 24 years [2]. The UCL complex is comprised of three components: the anterior oblique, posterior oblique, and transverse bands. The anterior band is most commonly injured [3]. Currently, treatment ranges from conservative to surgical intervention pending degree of injury with a growing trend towards conservative approaches.

UCL tears typically present with symptoms such as pain on the medial side of elbow, reduced throwing velocity and accuracy, and sometimes a “pop” or tearing sensation during an athlete's throwing motion. Athletes may also experience elbow instability, leading to difficulty performing their sport-related activities [4].

UCL tears can be classified as grade 1, grade 2, and grade 3 injuries. Grade 1 tears are low-grade injuries that consist of edema around the ligament [4]. Grade 2 tears are medium-grade injuries, with disruption to some of the ligament, and grade 3 tears are high-grade injuries that consist of full ligament disruption [4,5]. Low- and medium-grade tears can typically be managed non-operatively with relative rest and then a progressive throwing program over the course of a few months [6].

Typical conservative, non-operative, management consists of pain control with non-steroidal anti-inflammatory medications, relative rest and rehabilitation [1]. Return to play for non-operative cases varies anywhere from 42-84% for those following a rehabilitation protocol [6]. Platelet-rich plasma (PRP) has gained traction as a treatment option in the management of musculoskeletal conditions, due to its demonstrated ability in in vitro studies to increase proliferation of ligament cells and collagen creation [7]. PRP uses an individual’s blood to harvest a concentrated injectate of platelets and growth factors. Thus far, there have been some case reports documenting the utility of PRP being used to successfully treat low- and medium-grade UCL tears, however, not high-grade tears [8]. In prior studies, use of PRP to treat partial tears resulted in an 88% success rate in returning athletes to play after an average of 12 weeks [6].

Those who sustain a high-grade tear upon injury as well as those who fail conservative management of low- and medium-grade tears are candidates for operative repair. The current standard surgical treatment for athletes with a ruptured, or high-grade, UCL tear looking to return to play is UCL reconstruction also known as “Tommy John” surgery. This procedure was initially developed by Dr. Frank Jobe, MD in 1974 and since its advent has significantly improved the prognosis of many overhead throwers returning to competition following UCL injury [2]. Athletes who undergo surgical correction are typically unable to return to competitive play until 12-15 months post-op [1].

This case explores the utility of a minimally invasive approach including PRP injection for a high-grade UCL tear.

## Case presentation

Case history

An 18-year-old right-hand-dominant competitive high school baseball pitcher presented for evaluation of right elbow pain. Over the course of the past 18 months, the pain had progressively worsened. Notably, the pain's intensity varied based on the type of pitch thrown and was most pronounced during fastball deliveries, reaching speeds of up to 90 miles per hour. As a starting pitcher with a rotation every five days, he experienced pain after completing three innings. Importantly, while the pain did not impact his pitching speed, it did influence his choice of pitches. The discomfort was localized to the inner aspect of the right elbow. Notably, the patient maintained consistent pitching mechanics and had not pursued any form of physical therapy before seeking medical attention. He denied experiencing any radiation of pain, numbness, or weakness in the right arm. Furthermore, his medical history was unremarkable, with no previous injuries, surgeries, or instances of trauma.

Physical exam

**Figure 1:**
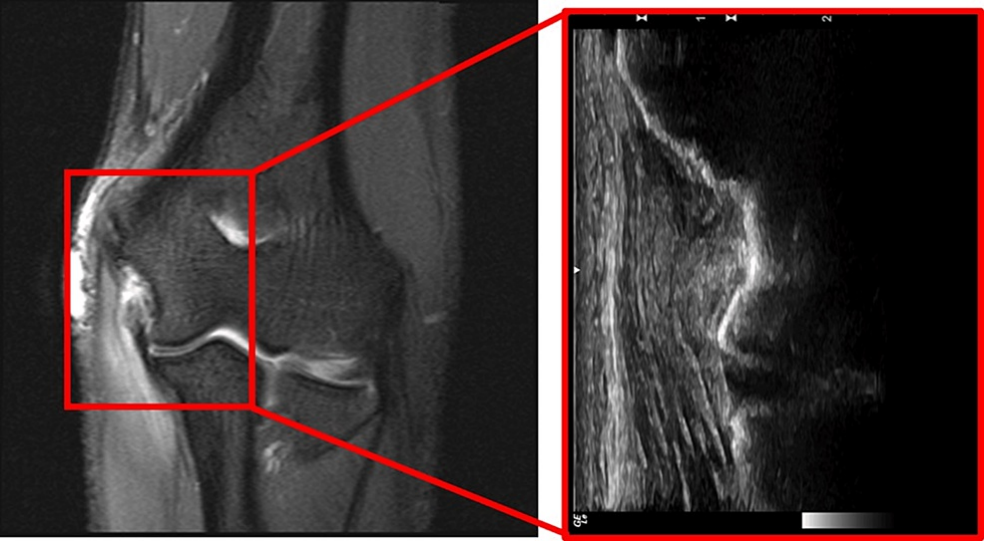
Magnetic Resonance Imaging and Ultrasound of the right elbow. Magnetic resonance imaging (MRI) demonstrates a near full-thickness, at least high-grade partial, tearing of the anterior bundle proximal portion of the ulnar collateral ligament (UCL) attachment on the distal aspect of the medial epicondyle. The zoomed image is from the associated diagnostic ultrasound which demonstrates hypoechoic thickening and loss of normal ligamentous echotexture involving the proximal portion of the anterior band of the ulnar collateral ligament.

The patient's posture was consistent with their age and gender, showing no significant deformities or abnormalities in alignment. There were no signs of swelling, redness, or muscle wasting. Upon palpation, tenderness was evident at the points corresponding to the origin and insertion of the ulnar collateral ligament in the right elbow. Dynamic stressing of the right UCL triggered pain. However, no outright instability of either the UCL or lateral collateral ligament (LCL) was observable. The patient's radial and ulnar pulses were graded as 2+, and sensation from C5 to T1 dermatomes was intact and symmetrical. Furthermore, strength testing of the right elbow yielded normal results. MRI demonstrated a near full-thickness, at least high-grade partial, tearing of the anterior bundle proximal portion of the UCL attachment on the distal aspect of the medial epicondyle (Figure 1). The zoomed image is from the associated diagnostic ultrasound which demonstrates hypoechoic thickening and loss of normal ligamentous echotexture involving the proximal portion of the anterior band of the UCL (Figure 1).

Treatment and patient course

The MRI of the right elbow unveiled a high-grade partial tear originating from the UCL. A dynamic ultrasound assessment of the right elbow revealed UCL stability. Opting for a non-operative approach, the patient underwent an ultrasound-guided PRP injection into the affected UCL. Subsequently, the patient engaged in an intensive three-month physical therapy regimen, encompassing graduated exercises and progressive simulations of throwing.

**Figure 2:**
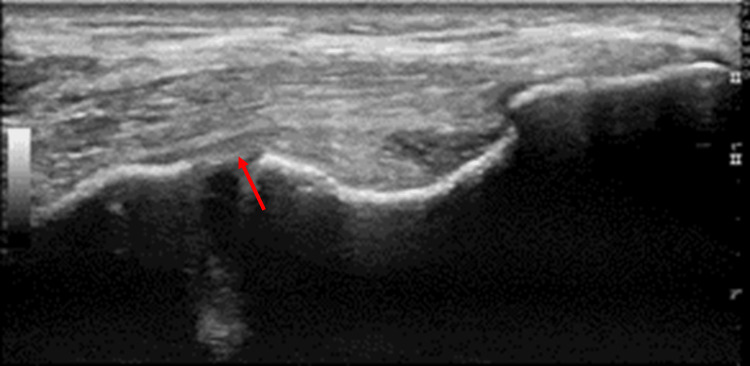
Post-treatment 12-week ultrasound. Demonstrates improved ligamentous echotexture and a healthy-appearing ulnar collateral ligament (arrow).

At the initial six-week follow-up, the patient reported a notable reduction in subjective pain. A limited ultrasound at this point indicated an enhancement in ligamentous echotexture and an expansion in joint space. Accordingly, an additional six weeks of therapy were recommended. Upon evaluation at the twelve-week mark, the patient's pain had fully subsided. The limited ultrasound imagery at this juncture displayed a healthy appearance of the UCL, with sustained joint expansion (Figure 2). Following the completion of the 12-week therapy protocol, the patient embarked on throwing exercises from the pitcher's mound, commencing at 50% effort and progressively advancing to full-strength throwing.

## Discussion

The presented case illustrates an intriguing instance of a high-grade partial UCL tear in a young baseball pitcher managed successfully through non-operative means. UCL tears are a considerable concern among throwing athletes, primarily due to the high valgus stresses imposed on the elbow during overhead activities. While surgical intervention has traditionally been favored for high-grade tears, there is an emerging interest in conservative approaches to manage UCL injuries, especially for partial tears [6]. The patient's responsiveness to non-operative management was notable in regard to decreased pain, increased function, improvement in the appearance of the ligament and return to activity. In comparison to traditional surgical intervention, his recovery was much faster requiring only 12 weeks of therapy before returning to pitching at full speed. In comparison, following surgical repair, a thrower is unable to begin a throwing protocol until four to five months post-op.

Ultrasound-guided UCL PRP injection, a minimally invasive technique, has gained traction due to its potential to facilitate tissue healing and repair. PRP has demonstrated success in returning pitchers to throwing quickly and successfully 73-96% of the time following medium-grade UCL tears or UCL insufficiency [7]. Typically in high-grade UCL tears, PRP has shown little benefit, with a 12.5% success rate while low-grade I and II tears are more frequently successfully treated with PRP and physical therapy [8]. The combination of PRP with a structured physical therapy program tailored to the individual's needs likely played a pivotal role in the patient's recovery.

Dynamic ultrasound imaging provided a real-time assessment of the ligament's healing process and joint widening. This underscores the value of such imaging modalities in monitoring the response to treatment, potentially reducing unnecessary invasive interventions [1,2]. This also allows for earlier referral for surgical intervention in the event healing is not demonstrated during ultrasound evaluation. Dynamic testing can be performed by applying a valgus stress to the elbow while maintaining visibility of the UCL on ultrasound. Here, the UCL can be assessed for any changes within the ligament itself (hypoechoic areas), as well as increased laxity in the UCL. The patient's ability to resume throwing activities without pain and with a healthy-appearing UCL on ultrasound indicates that conservative management can yield satisfactory outcomes.

While this case is encouraging, individualized treatment strategies remain crucial. Factors such as tear severity, patient compliance, and the specific demands of the sport must be considered [9]. Additionally, it is important to consider the potential complications that may be associated with use of PRP injections. This includes but may not be limited to onset of ulnar nerve irritation [10]. Further research is warranted to determine the optimal protocols for conservative management, including the role of PRP injections, the length of time PRP resolves symptoms, the rate of re-tearing of the UCL after PRP injection, and tailored rehabilitation programs in various grades of UCL tears.

## Conclusions

This case report sheds light on the potentially successful opportunity for non-operative management of a high-grade partial UCL tear in a competitive baseball pitcher. The patient's significant improvement in pain, coupled with objective findings of structural healing and joint widening on dynamic ultrasound, demonstrates the potential effectiveness of guided PRP injection and intensive physical therapy in promoting tissue repair and functional recovery. While surgical intervention remains a common approach, this case underscores the importance of considering conservative strategies, especially for partial UCL tears. Tailored rehabilitation programs and close monitoring through dynamic imaging modalities can contribute to positive outcomes. Nonetheless, a personalized approach, accounting for tear severity, patient compliance, and athletic demands, remains paramount in determining the most appropriate management pathway. Further research is necessary to refine non-operative protocols for UCL injuries and to establish their broader applicability within the spectrum of throwing-related sports injuries.
